# Drp1 inhibition attenuates neurotoxicity and dopamine release deficits *in vivo*

**DOI:** 10.1038/ncomms6244

**Published:** 2014-11-05

**Authors:** Phillip M. Rappold, Mei Cui, Jonathan C. Grima, Rebecca Z. Fan, Karen L. de Mesy-Bentley, Linan Chen, Xiaoxi Zhuang, William J. Bowers, Kim Tieu

**Affiliations:** 1 Department of Environmental Medicine, Center for Translational Neuromedicine, University of Rochester School of Medicine, 575 Elmwood Avenue, Rochester, New York 14642, USA; 2 Department of Clinical Neurobiology and Institute of Translational and Stratified Medicine, Plymouth University, John Bull Building, Plymouth PL6 8BU, UK; 3 Department of Pathology and Laboratory Medicine, University of Rochester Medical Center, 601 Elmwood Avenue, Rochester, New York 14642, USA; 4 Department of Neurobiology, University of Chicago, 947 E. 58th Street, Chicago, Illinois 60637, USA; 5 Department of Neurology, Center for Neural Development and Disease, University of Rochester Medical Center, 601 Elmwood Ave., Rochester, New York 14642, USA

## Abstract

Mitochondrial dysfunction has been reported in both familial and sporadic Parkinson’s disease (PD). However, effective therapy targeting this pathway is currently inadequate. Recent studies suggest that manipulating the processes of mitochondrial fission and fusion has considerable potential for treating human diseases. To determine the therapeutic impact of targeting these pathways on PD, we used two complementary mouse models of mitochondrial impairments as seen in PD. We show here that blocking mitochondrial fission is neuroprotective in the PTEN-induced putative kinase-1 deletion (*PINK1*^*−/−*^) and 1-methyl-4-phenyl-1,2,3,6-tetrahydropyridine mouse models. Specifically, we show that inhibition of the mitochondrial fission GTPase dynamin-related protein-1 (Drp1) using gene-based and small-molecule approaches attenuates neurotoxicity and restores pre-existing striatal dopamine release deficits in these animal models. These results suggest Drp1 inhibition as a potential treatment for PD.

## Abstract (web summary)

Mitochondrial dysfunction has been associated with Parkinson’s disease but effective therapies targeting this pathway are yet to be developed. Here the authors show that inhibition of the mitochondrial fission protein Drp-1 using genetic or small-molecule approaches in mouse models of the disease, leads to improvements in the pathology.

## Graphical abstract

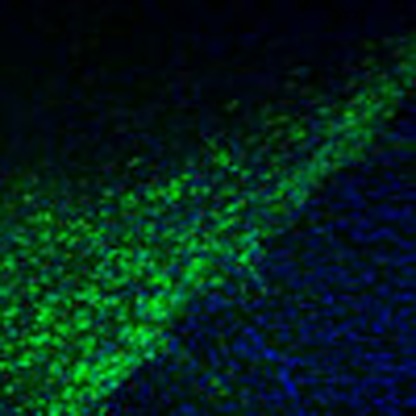

Parkinson’s disease (PD), a neurological disorder characterized by degeneration of the nigrostriatal dopamine (DA) pathway, currently has no established neuroprotective or neurorestorative treatments. The link between mitochondrial dysfunction and PD has been proposed since the discovery of parkinsonism in humans caused by 1-methyl-4-phenyl-1,2,3,6-tetrahydropyridine (MPTP)-contaminated synthetic meperidine. However, it was the discovery of mutations in PTEN-induced putative kinase-1 (*PINK1*), as a cause of autosomal recessive PD that established a strong genetic link between PD and mitochondria. Other PD-linked mutations such as α-synuclein, LRRK2, parkin and DJ-1 have also been demonstrated to adversely affect mitochondrial function. These recent genetic studies also have uncovered mitochondrial dynamics (fission/fusion/movement) as a potential therapeutic target for PD^1^,^2^,^3^,^4^,^5^.

Mitochondria are dynamic and they undergo frequent changes in shape, size, number and location. These alterations can be affected by mitochondrial morphology, which in turn, is controlled mainly by the processes of fission and fusion. In mammals, the outer mitochondrial proteins mitofusins (Mfn1 and Mfn2) and the inner mitochondrial protein optic atrophy 1 (OPA1) are responsible for mitochondrial fusion. Fission on the other hand requires the recruitment of dynamin-related protein 1 (Drp1) from the cytosol to the outer mitochondrial membrane by Fission-1 (Fis1) and mitochondrial fission factor (Mff). This process produces multiple smaller mitochondria, which are more motile within the cell. Fission is also important for mitochondrial quality^2^,^6^. In contrast to fission, fusion results in larger mitochondria, which could offer a larger ATP supply and also facilitate inter-mitochondrial exchange of substrates and mitochondrial DNA (mtDNA) leading to functional complementation between defective and functional mitochondria^2^,^7^. The dynamic relationship between fission and fusion also plays a role in regulating cell death through cytochrome *c* release. Consequently, a balance of fusion and fission is crucial not only to mitochondrial morphology, but also to cell viability and synaptic function.

Synaptic dysfunction has gained attention in recent years as an early pathology that arises before neurodegeneration in different neurological disorders, including PD^8^,^9^. PD genetic models demonstrate that parkin, DJ-1, PINK1, α-synuclein and LRRK2 play a role in striatal presynaptic DA release. However, the mechanisms by which presynaptic dysfunction is caused by these mutations remain largely unknown. Relevant to the present study, mitochondria play a crucial role in presynaptic function^4^,^10^,^11^,^12^, consistent with high energy demand by synaptic terminals to maintain synaptic membrane potential, restore ionic gradients following synaptic transmission and reload synaptic vesicles with neurotransmitters. In addition, presynaptic mitochondria play a major role in sequestration of cytosolic Ca^2+^ during normal neurotransmission^10^,^11^. Further supporting the role of mitochondria in presynaptic release, pharmacological and genetic blockade of mitochondrial function have been demonstrated to depress synaptic transmission^13^. Taken together, restoring mitochondrial function may therefore attenuate synaptic failure and neurodegeneration in PD.

Previous studies including our own reported that blocking Drp1 function was sufficient to attenuate mitochondrial dysfunction and neurotoxicity in PD cell culture models^14^,^15^,^16^,^17^,^18^. However, to date, the *in vivo* significance of blocking this mitochondrial fission protein in animal models of nigrostriatal dysfunction has remained undetermined. In this study, we utilized complementary mouse models with mitochondrial impairments in the nigrostriatal system: *Pink1-*knockou*t* (*Pink1*^*−/−*^) mice represent a human disease relevant target with impairments in mitochondrial complex I function and evoked striatal DA release^19^,^20^,^21^,^22^. The neurotoxin MPTP-treated mice provide a model of complex I inhibition with nigrostriatal neurotoxicity^23^. Because both mitochondrial fission and fusion are important for cellular homeostasis, we assessed both manipulation strategies. We report here that blocking mitochondrial fission is beneficial in these animal models.

## Results

### Adeno-associated virus (AAV2)-mediated expression of Drp1-K38A and Fis1 in DA neurons

**Figure 1:**
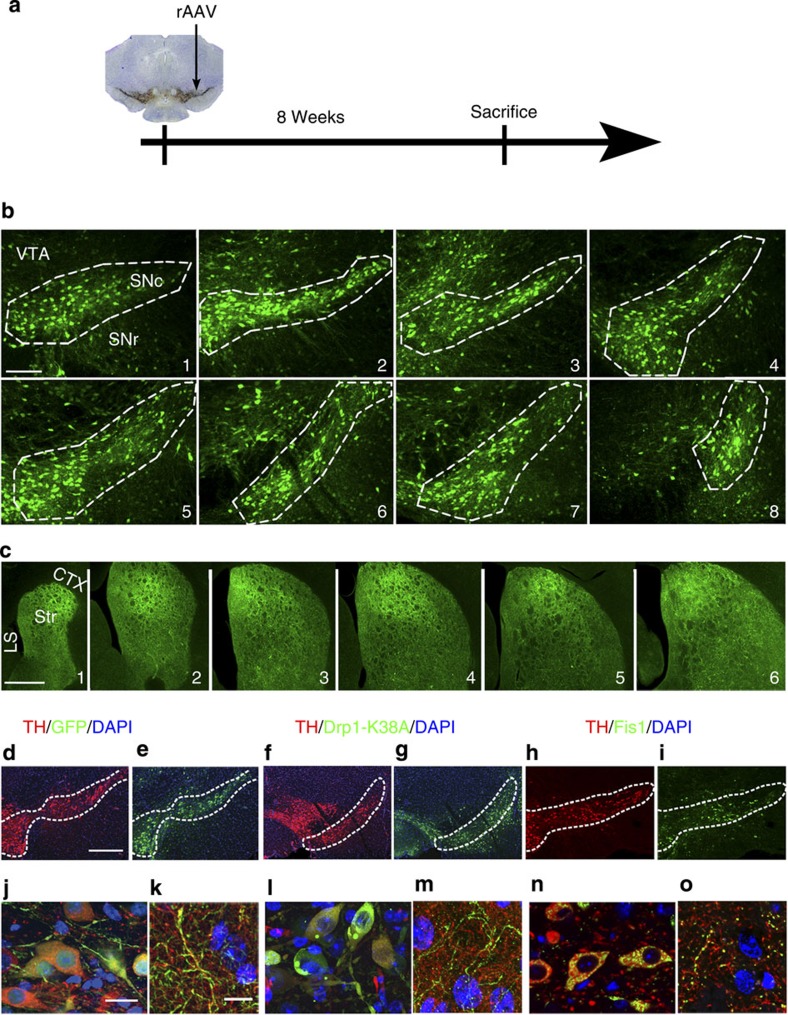
rAAV2-mediated gene expression in the nigrostriatal pathway. (**a**) Schematic illustrating stereotactic infusion of rAAV2 (5 × 10^9^ viral particles) slightly dorsal to the substantia nigra pars compacta (SNc, arrows). (**b**) Expression pattern of GFP is illustrated in serial sections of SNc from its most rostral (**b**1) to caudal (**b**8) boundaries after 8 weeks of rAAV2-eGFP gene delivery. (**c**) Levels of rAAV2-mediated GFP expression in nigral axonal projections throughout the rostral (**c**1) to caudal (**c**6) extent of the striatum, and in adjacent brain structures such as the cortex (CTX) and lateral septum (LS). Immunofluorescent signals of Drp1-K38A (a dominant negative mutant of Drp1), Fis1 and GFP control in nigral DA cell bodies (**e**,**g**,**i**,**j**,**l**,**n**) and their striatal terminals (**k**,**m**,**o**) 8 weeks after supranigral stereotactic delivery of 5 × 10^9^ rAAV2 particles. Scale bars (**b**1–**b**8), 400 μm, (**c**1–**c**6) 1 mm, (**d**–**i**) 400 μm, (**j**,**l**,**n**) 20 μm, (**k**,**m**,**o**) 10 μm.

We previously demonstrated that the Drp1-dominant negative Drp1-K38A mutant was protective in stable dopaminergic neuronal cell model of PD^14^. This genetic approach has also been extensively used to block Drp1 function in various *in vitro* disease models^15^,^16^,^24^. In the present study, we performed supranigral injections of recombinant adeno-associated virus (rAAV2) to deliver Drp1-K38A to block mitochondrial fission, Fis1 to promote fission or enhanced green fluorescent protein (eGFP) as a control (Fig. 1a). Using convection-enhanced gene delivery technique, robust protein expression was detectable 8 weeks later throughout the substantia nigra pars compacta (SNc) and striatum from rostral to caudal regions (Fig. 1b–o). Transduction efficiency in nigral DA neurons was calculated as we previously described by quantifying tyrosine hydroxylase (TH)-positive neurons transduced with eGFP^25^ (Fig. 1b). Both *PINK1*^*+/+*^ and *PINK1*^*−/−*^ mice had comparable transduction rates (71.3% ±0.05 versus 74.0% ±0.05, respectively. Data represent mean±s.e.m., *n*=3 mice per group).

### Drp1-K38A and Fis1 alter mitochondrial morphology *in vivo*

**Figure 2:**
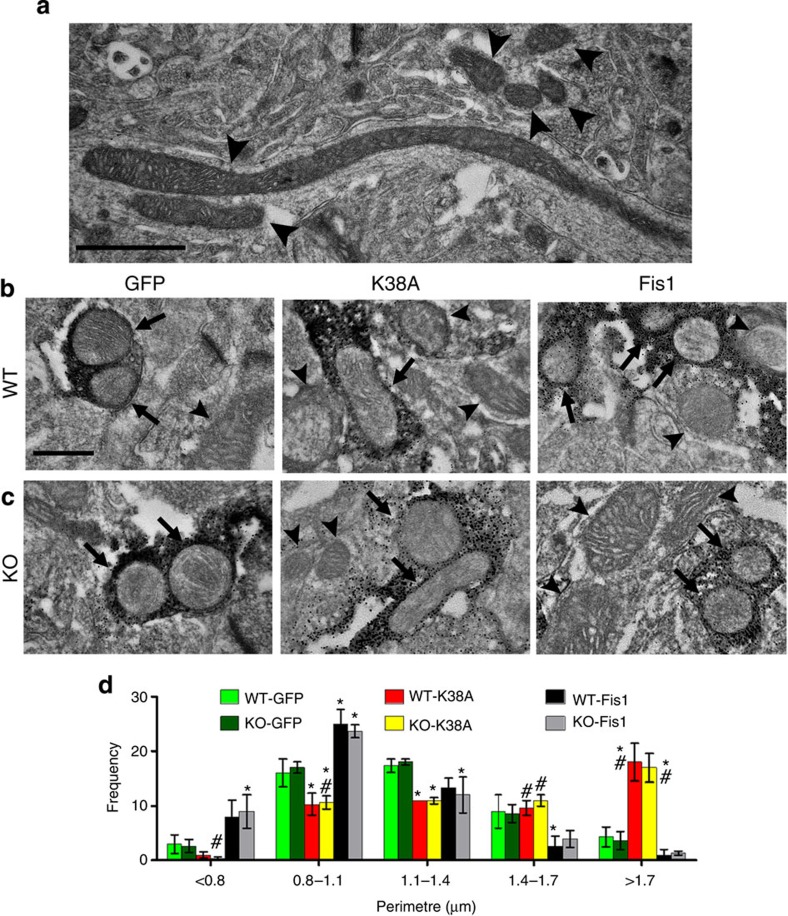
Morphological analyses of mitochondria in striatal terminals. (**a**) Electron microscopy (EM) shows the size and shape of mitochondria in the striatum ranging from small to highly elongated morphology. For ultrastructural analyses of mitochondria in striatal DA axonal terminals, immuno-EM was performed in coronal striatal sections from ~1-year-old *Pink1*^+/+^ (WT, **b**) and *Pink1*^*−*/*−*^ (KO, **c**) littermates transduced with eGFP control, Drp1-K38A or Fis1. Arrows indicate tyrosine hydroxylase (TH)-positive axonal terminals containing mitochondria, whereas arrowheads indicate those in TH-negative structures. Quantitative measurements of mitochondria were expressed as perimeter (**d**). Data represent mean±s.e.m. of three animals with 50 clearly identifiable mitochondria (cristae and/or double membrane) randomly and blindly selected per mouse, grouped into different size bins and analysed using two-way analysis of variance (Bin <0.8 μm: *genotype*: *F*_1,12_=0.00, *P*=1.00; AAV *constructs*: *F*_2,12_=8.33, *P*<0.01; Bin 0.8–1.1 μm: *genotype*: *F*_1,12_=0.00, *P*=1.00; *AAV constructs*: *F*_2,12_=27.07, *P*<0.001; Bin 1.1–1.4 μm: *genotype*: *F*_1,12_=0.00, *P*=1.00; *AAV constructs*: *F*_2,12_=9.29, *P*<0.01; Bin 1.4–1.7 μm: *genotype*: *F*_1,12_=0.27, *P*=0.61; *AAV constructs*: *F*_2,12_=8.02, *P*<0.001; Bin >1.7 μm: *genotype*: *F*_1,12_=0.07, *P*=0.80; *AAV constructs*: *F*_2,12_=35.16, *P*<0.001). Pairwise comparison was performed using Newman–Keuls *post-hoc* test. *P<0.05 compared with the GFP group, ^*#*^*P*<0.05 compared with the Fis1 group, respectively, within each genotype. Scale bars (**a**), 1 μm, (**b**,**c**), 400 nm.

After 8 weeks to allow sufficient time for protein expression in the nigral DA neurons, we determined the effects of fission/fusion proteins on mitochondrial morphology. Given the heterogeneous mitochondrial morphology observed between cell types (Fig. 2a), we performed immuno-electron microscopy to specifically carry out analyses in striatal DA terminals (Fig. 2b,c, Supplementary Fig. 1), a site where mitochondria control presynaptic release of DA in the nigrostriatal pathway. Quantitative morphological analysis confirmed that as compared with the eGFP group, there was a larger proportion of elongated mitochondria in mice expressing Drp1-K38A and an increased fraction of smaller mitochondria in the Fis1 group (Fig. 2d), consistent with the role of blocking and promoting fission of these proteins, respectively. Two-way analysis of variance analyses indicate these alterations were Pink1-independent as no difference between genotypes was detectable in the absence or presence of these constructs, suggesting mitochondrial morphology in DA terminals of mice with germline deletion of *Pink1* is not altered, and furthermore, mitochondrial dysfunction in *Pink1*^*−*/*−*^ mice^20^,^21^ is not necessarily linked to mitochondrial fragmentation as seen in cell culture models with transient or acute loss of PINK1 function^14^,^15^,^16^,^26^.

### Drp1 inhibition restores DA release in *Pink1* ^*−/−*^ mice

**Figure 3:**
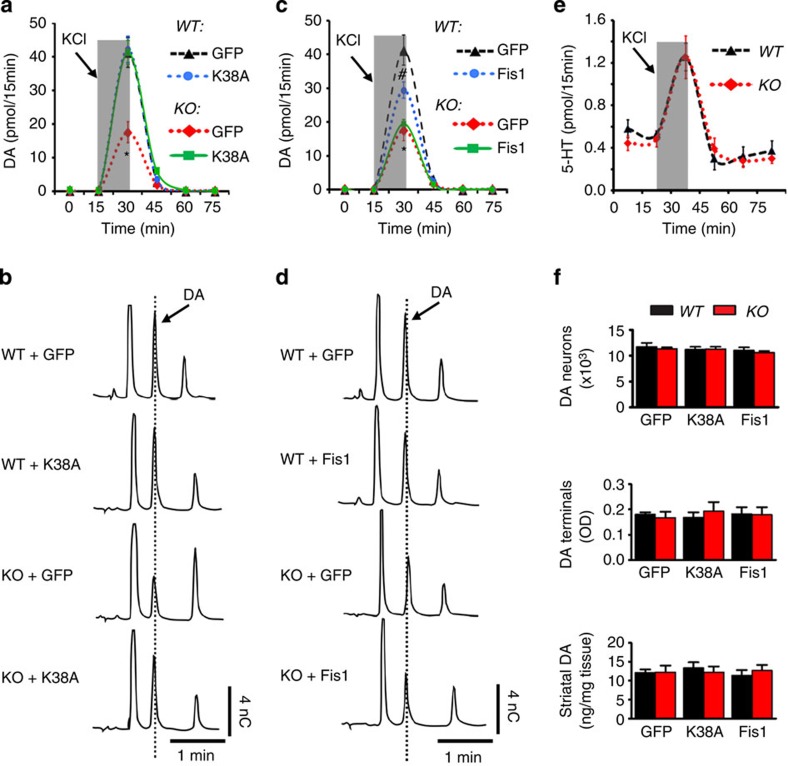
Drp1-K38A restores synaptic release of DA in *Pink1*^*−/−*^ mice. Approximately 1-year-old *Pink1*^*−*/*−*^ (KO) and *Pink1*^+/+^ (WT) littermates were transduced with eGFP-, Drp1-K38A- (**a**,**b**) or Fis1 (**c**,**d**)-expressing AAV2 vectors for 8 weeks before *in vivo* microdialysis was performed in freely moving mice. To evoke depolarization-induced release of DA, a total 240 nmol KCl in isotonic aCSF was delivered through the probe over a 15-min period (shaded box). Striatal dialysates were collected every 15 min and analysed simultaneously for DA and serotonin (**e**) levels using HPLC. Representative HPLC chromatograms of the 30-min fractions of **a** and **c** are shown in **b** and **d**, respectively. Areas under the curve of **a** and **c** were generated using GraphPad Prism and analysed by two-way analysis of variance (genotype × treatment: *F*_*2*,17_=8.63, *P*=0.003; *n*=3–5 mice per group), followed by Newman–Keuls *post-hoc* test. (**a**): *P<0.001 KO-GFP versus WT-GFP or versus KO-K38A. (**c**): *P<0.001 KO-GFP versus WT-GFP, ^#^*P*=0.012 (WT-Fis1 versus WT-GFP) or ^#^*P*=0.041 (WT-Fis1 versus KO-Fis1). (**f**) After microdialysis, brains were removed and processed for stereological cell counts of DA neurons, striatal terminal density and total striatal DA content.

Mitochondria from *Pink1*^*−*/*−*^ mice exhibit respiratory dysfunction^20^,^21^ and evoked DA release is impaired in acute striatal slices of these animals^22^. These observations are consistent with the crucial role of mitochondria in presynaptic release^4^,^10^,^11^. We asked whether this deficit in DA release also occurred *in vivo*, and if so, whether blocking or promoting fission would rectify this defect. To this end, we used *in vivo* microdialysis to assess depolarization-induced DA overflow in the striatum of freely moving *Pink1*^*−/−*^ and *Pink1*^*+/+*^ littermates. *Pink1*^*−/−*^ mice exhibited significantly reduced DA overflow compared with *Pink1*^*+/+*^ controls (Fig. 3a–d, Table 1). Simultaneous quantification of serotonin (Fig. 3e) in these dialysates suggests this deficit was specific to DA. Of note, impaired DA overflow in *Pink1*^*−/−*^ mice was not a result of nigrostriatal damage (Fig. 3f) or increased dopamine transporter (DAT) activity^22^. Our observation here provides *in vivo* evidence of impaired exocytotic DA release in *Pink1*^*−/−*^ mice. However, 8 weeks after receiving Drp1-K38A (Fig. 3a,b, Table 1), but not Fis1 (Fig. 3c,d Table 1), a restoration of evoked DA overflow was achieved in *Pink1*^*−/−*^ mice, suggesting blocking, but not promoting fission, is beneficial. Furthermore, in *Pink1*^*+/+*^ littermates, Drp1-K38A had no detectable effect, whereas Fis1 significantly reduced DA release. Because no changes in the number of nigral DA neurons, striatal DA terminals and total DA content occurred (Fig. 3f), the alterations in DA release were related to function, not structure of the nigrostriatal pathway. Together, these results indicate that blocking mitochondrial fission is capable of ameliorating pre-existing DA synaptic dysfunction in *Pink1*^*−/−*^ mice. In addition, in contrast to Fis1, Drp1-K38A does not appear to negatively impact DA release in *Pink1*^*+/+*^ mice.

**Figure 4:**
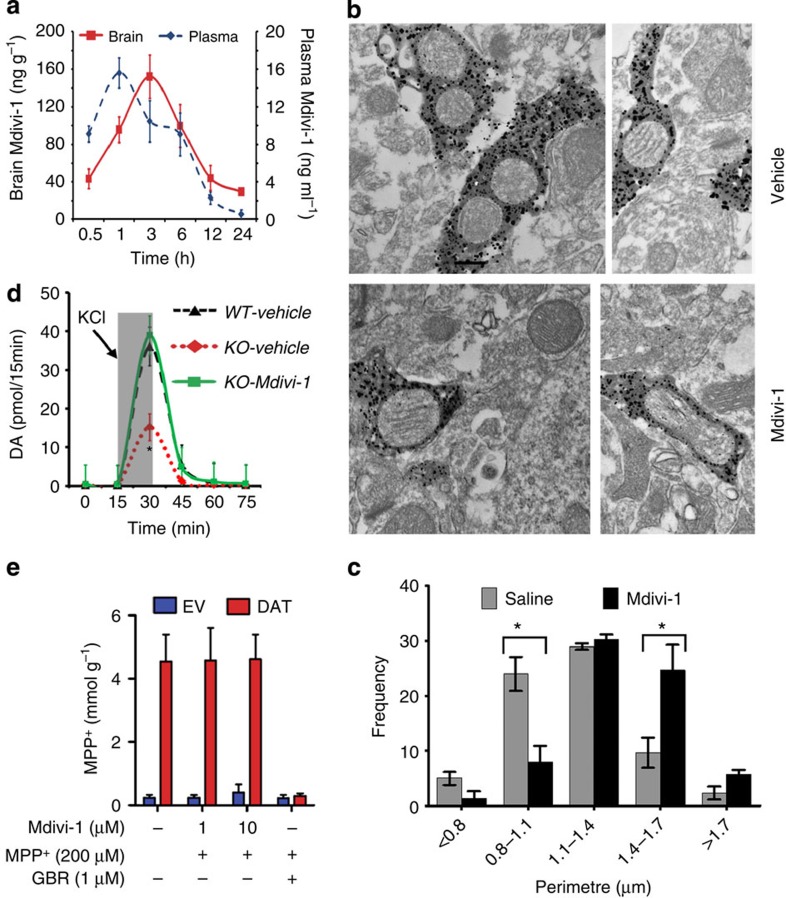
Mdivi-1 restores synaptic release of DA in *Pink1*^*−/−*^ mice. To demonstrate that Mdivi-1 is permeable to the blood–brain barrier, C57Bl/6 mice were injected i.p. with mdivi-1 (20 mg kg^−1^) and killed at indicated time points (**a**). Mdivi-1 levels in brain and plasma samples were quantified using HPLC (*n*=4 per group). (**b**,**c**) After receiving twice daily injection of mdivi-1 (20 mg kg^−1^) for 3 days, mitochondrial morphology in striatal dopaminergic terminals was quantified using immuno-electron microscopy as described in Fig. 2. Using this same dosage regimen, ~1-year-old *Pink1*^*−*/*−*^ and *Pink1*^+/+^ received twice daily i.p. injection of mdivi-1 for 3 days followed by *in vivo* microdialysis (**d**), *n*=4–5 per group. *P=0.037 (WT-vehicle versus KO-vehicle) and *P=0.043 (KO-vehicle versus KO-mdivi-1 groups). (**e**) To assess the effect of mdivi-1 on DAT function, uptake of MPP^+^ into stable cells overexpressing DAT or empty vector control was performed in the presence or absence of mdivi-1 for 30 min, followed by HPLC measurement of MPP^+^. GBR was used as a control DAT inhibitor. *n*=4 independent experiment, each with triplicates.

As a complementary approach to Drp1-K38A, we turned to the small molecule termed Mitochondrial Division Inhibitor-1 (Mdivi-1)^27^. This Drp1 inhibitor has been demonstrated to be protective by blocking mitochondrial fission in cell culture models^14^,^27^,^28^,^29^ as well as in mouse models of renal^30^, cardiac^31^,^32^, brain ischemic damage^28^,^29^ and rat neuropathic pain^33^. We first assessed whether this molecule crossed the blood–brain barrier because this property had not been demonstrated in previous studies. Using a newly developed high-performance liquid chromatography (HPLC) method, peak plasma levels of mdivi-1 were seen 1 h after intraperitoneal (i.p.) injection, followed by peak brain levels 2 h later, confirming this lipophilic molecule is permeable to the blood–brain barrier (Fig. 4a). Based on the estimated half-life of mdivi-1 in the brain (~8.9 h), we chose a twice daily dosing regimen in the subsequent studies. Similar to Drp1-K38A, more mitochondrial elongation was detectable in mice treated with mdivi-1 twice daily for 3 days compared with the group that received vehicle control (Fig. 4b,c). Furthermore, this small molecule also restored evoked DA overflow in *Pink1*^*−/−*^ mice (Fig. 4d, Table 2). As subsequently illustrated in Fig. 7a,b, mdivi-1 did not affect DA outflow in wild-type mice. To eliminate the possibility that this enhanced DA overflow in *Pink1*^*−/−*^ mice was not due to mdivi-1 interfering with DA reuptake, we utilized stable cells overexpressing DAT in the presence of its substrates 1-methyl-4-phenylpyridinium (MPP^+^; Fig. 4e). Mdivi-1 did not have an effect on DAT function up to 10 μM. In contrast, 1 μM of GBR 12909 (a DAT inhibitor) blocked more than 90% of DAT transport activity. Collectively, these results indicate mdivi-1 is capable of ameliorating the pre-existing DA release deficits in *Pink1*^*−/−*^ mice.

### Drp1 inhibition attenuates dopaminergic neurotoxicity

**Figure 5:**
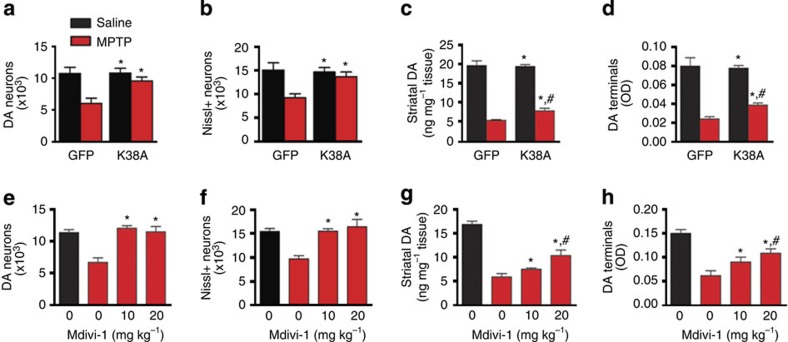
Drp1-K38A and Mdivi-1 prevent MPTP-induced neurotoxicity. (**a**–**d**) Approximately 10-week-old C57Bl/6 mice were transduced with rAAV2 as described above. Eight weeks after gene delivery, mice were injected once daily for 5 days with MPTP (20 mg kg^−1^, i.p.) or saline. Seven days after the last injection, mice were assessed for nigrostriatal integrity by performing stereological cell counts of nigral DA neurons (**a**) and of total nigral Nissl-positive neurons (**b**), striatal DA terminal density (**c**) and total striatal DA content (**d**). *n*=3–6 per group, two-way analysis of variance (ANOVA) followed by Newman–Keuls *post-hoc* test. *P<0.05 compared with the GFP-MPTP group; ^#^*P*<0.05 compared with the Drp1-K38A-saline group. (**e**–**h**) C57Bl/6 mice receiving MPTP or saline were injected i.p. with Mdivi-1 (20 mg kg^−1^) or vehicle twice daily until day the mice were killed. Seven days after the last MPTP injection, neuropathology was assessed. *n*=4–7 per group, analysed by one-way ANOVA followed by Newman–Keuls *post-hoc* test. *P<0.05 compared with animal receiving MPTP without mdivi-1; ^#^*P*<0.05 compared with animals receiving saline.

To determine the impact of Drp1 inhibition against neurotoxicity in the nigrostriatal pathway, ~10-week-old CB7Bl/6 mice received gene delivery as described above for 8 weeks before MPTP injections. Drp1-K38A significantly attenuated damage induced by MPTP (Fig. 5a–d). To further support that Drp1 inhibition protected against cell loss, rather than increasing the expression of the phenotypic marker tyrosine hydroxylse (TH), we also performed stereological counting of Nissl-positive neurons in the SNc (Fig. 5b). These quantitative data indicate that, first, at this time point (7 days after the last MPTP injection), there was a significant loss of nigral neurons, not a downregulation of TH phenotype^34^, consistent with our previous validation^35^. Second, Drp1-K38A prevented cell loss, and thus did not merely promote re-expression of the TH marker. Consistent with the effect of this Drp1 dominant negative, twice daily injections of mdivi-1 to adult C57Bl mice during MPTP treatment also prevented neurotoxicity of DA neurons, striatal terminals and total striatal DA content (Fig. 5e–h). Of note, Mdivi-1 did not interfere with the brain levels of 1-methyl-4-phenylpyridium (MPP^+^) after MPTP injection, as striatal levels of this active metabolite of MPTP were comparable between mice receiving mdivi-1 (10.73±0.38 μg g^−1^ tissue. Data represent mean±s.e.m., *n*=4.) and vehicle control (10.65±1.14 μg g^−1^ tissue. Data represent mean±s.e.m., *n*=5.). Together, these results demonstrate that blocking Drp1 function *in vivo* either through genetic or small-molecule approach is neuroprotective in a mouse model with damage in the nigrostriatal pathway.

### Drp1 inhibition improves DA release in MPTP pre-treated mice

**Figure 6:**
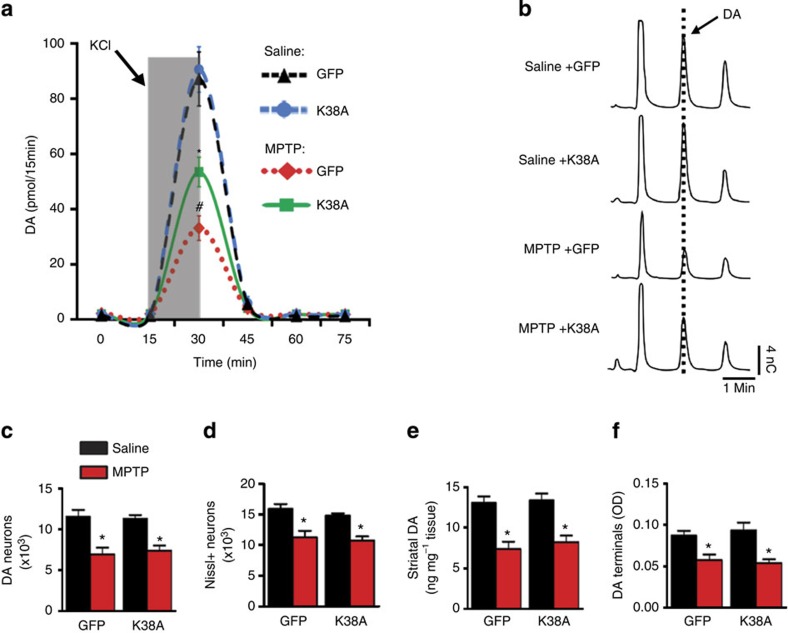
Drp1-K38A restores DA release in the MPTP pre-lesioned mice. (**a**) Approximately 10-week-old C57Bl/6 mice were injected with MPTP (20 mg kg^−1^, i.p. once daily for 5 days) or saline and 7 days after the last injection, rAAV2 was delivered to the SNc 8 weeks before *in vivo* microdialysis. KCl-evoked DA release was performed as described in Fig. 3. Data represent mean±s.e.m. (two-way analysis of variance: F_1,13_=11.89, *P*=0.004, *n*=4–5 mice per group, followed by Newman–Keuls *post-hoc* test, *P=0.001 (MPTP-K38A versus MPTP-GFP), ^#^*P*=0.003 (MPTP-K38A versus Saline-K38A). (**b**) Representative HPLC chromatograms of the 30-min fractions of **a**. After microdialysis, brains were removed and processed for stereological cell counting (**c**,**d**), total striatal DA content (**e**) and striatal DA terminals (**f**).

**Figure 7:**
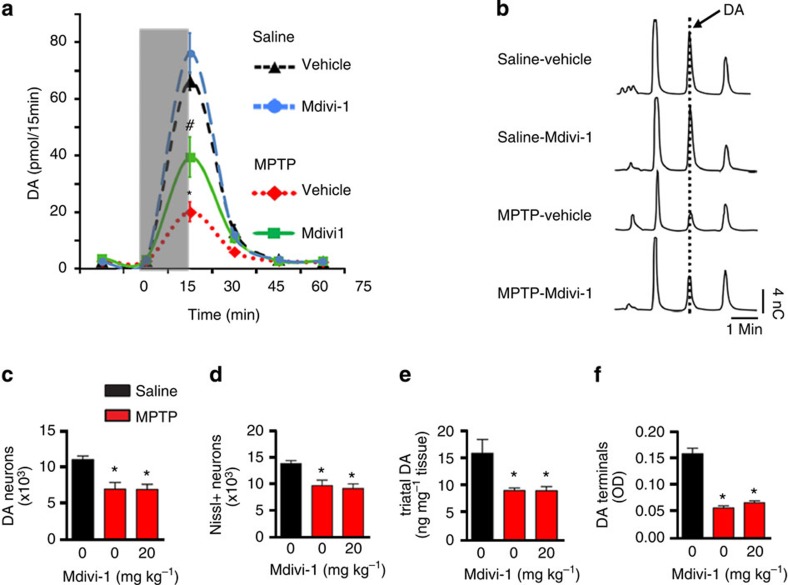
Mdivi-1 restores DA release in the MPTP pre-lesioned mice. (**a**) Seven days after the last MPTP injection as described in Fig. 6, mice received i.p. injections of mdivi-1 (20 mg kg^−1^ twice daily for 3 days) or vehicle control before *in vivo* microdialysis was performed and analysed by one-way analysis of variance. *n*=4–5 mice per group. *P=0.0013 (MPTP-vehicle versus MPTP-mdivi1), *P<0.001 (MPTP-mdivi1 versus saline-vehicle or Saline-mdivi1). (**b**) Representative HPLC chromatograms of the 30-min fractions of **a**. After microdialysis, brains were removed and processed for stereological cell counting (**c**,**d**), total striatal DA content (**e**) and striatal DA terminals (**f**).

Considering the substantial amount of nigrostriatal degeneration already present at the time of diagnosis in humans with PD, we aimed to more closely model this scenario and assessed the functional impact of blocking Drp1. To this end, mice received MPTP as described above, however, we delayed gene delivery until 7 days after the last injection to allow the lesion to form and stabilize before intervention. We hypothesized that among the remaining nigrostriatal neurons, there would exist a sizable dysfunctional fraction that could be ameliorated by promoting mitochondrial fusion—a process that could restore mitochondrial function through functional complementation between defective and functional mitochondria^2^,^7^. In MPTP pre-lesioned mice, both Drp1-K38A (Fig. 6a,b, Table 3a) and mdivi-1 (Fig. 7a,b, Table 3b) improved evoked DA overflow despite having no effects on nigrostriatal structure and total striatal content (Figs 6c,f and 7c–f). These observations are consistent with our hypothesis that the underlying dysfunctional cellular pathway is one capable of being rapidly restored, such as membrane potential, bioenergetic status and/or Ca^2+^ buffering capacity of mitochondria but not through restoring cell population and DA content mediated by neurogenesis or recovery of TH expression and function within this short time-frame.

## Discussion

Targeting mitochondrial dynamics as a potential therapeutic for neurodegenerative diseases has been highlighted in recent years^1^,^2^,^3^,^4^,^5^. Overall, it has been documented that either too much fission or fusion is not conducive to optimal mitochondrial function. Hence, such imbalances could negatively impact synaptic function and neuronal viability. In cell culture models, in addition to PD-linked mutations, neurotoxic molecules such as rotenone^36^, MPP^+^ (ref. 17), methamphetamine^37^ and 6-hydroxydopamine^18^, which are capable of damaging the nigrostriatal pathway, cause mitochondrial fission and neurotoxicity that can be attenuated by blocking mitochondrial fission or promoting fusion. Thus, perturbed mitochondrial dynamics may represent a shared mechanism that underlies both genetic and toxin-induced related PD. The present study utilizes both genetic and neurotoxic models of nigrostriatal dysfunction to determine the *in vivo* significance of targeting mitochondrial fission/fusion in PD.

*Pink1*^*−/−*^ mice were used to represent a human genetic model with impairments in mitochondrial complex I function and evoked striatal DA release^19^,^20^,^21^,^22^. The recognition that synaptic dysfunction is an early and likely pathogenic factor in neurodegenerative disorders^8^,^9^ and the much shorter lifespan in mice compared with humans have led to the view that such genetically engineered mice may model the early pre-clinical stages of PD^38^. In the present study, we observed that blocking mitochondrial fission via Drp1 inhibition rescues DA release deficits in *Pink1*^*−/−*^ mice. Although the role of PINK1 on mitochondrial fission/fusion remains a topic of debate, primarily due to conflicting observations between the published *Drosophila* and mammalian cell culture studies, insights into these discrepancies have recently been extensively discussed^3^,^39^. Briefly, loss of PINK1 function has been demonstrated to tip the balance of fission/fusion towards an overall pathogenic mitochondrial fission in mammalian immortalized cell models^14^,^15^,^16^,^26^. In *Drosophila* models, however, Pink1 has the opposite effect^40^,^41^,^42^. Potential explanations for this discrepancy include the time when morphological analysis is performed after a loss of PINK1 function occurs and different compensatory mechanisms between models. Because of space constraints, further detailed discussion on this topic can be found in these reviews^3^,^39^. Although additional studies are required to address this issue, it is crucial to move forward to mammalian animal models to address whether blocking or promoting mitochondrial fission is beneficial to a compromised nigrostriatal dopaminergic system. Our study is designed to shed light on this critical question. The lack of detectable changes in mitochondrial morphology in mice with germline deletion of *Pink1* in the current study is consistent with the argument that the effect of PINK1 on mitochondrial morphology is transient and acute^3^,^39^. Studies using primary neurons or fibroblasts from *Pink1*^*−/−*^ mice also do not display mitochondrial fragmentation^20^,^43^,^44^. Despite the inconsistent observations of changes in mitochondrial morphology, a loss of PINK1 function is consistently reported to result in mitochondrial dysfunction across various models, suggesting mitochondrial morphology is not necessarily linked to mitochondrial dysfunction induced by a loss of PINK1 function.

In the absence of mitochondrial fragmentation in *Pink1*^*−/−*^ mice, the intriguing question is why does promoting mitochondrial fusion via blocking Drp1 function restored DA release in these animals? It has been established that within a single cell, wild-type mtDNA and mutant variants can co-exist (heteroplasmy) and when the damaged load exceeds a threshold of >60%, mitochondrial dysfunction occurs^45^,^46^. By promoting exchanges of mitochondrial contents and DNA between functional and defective mitochondria within heteroplasmic cells, mitochondrial fusion can dilute defective mitochondria and attenuate their negative impact through functional complementation^2^,^7^. Loss of PINK1 function has been reported to result in mtDNA mutations and defects in the electron transport chain in humans and experimental models^47^,^48^. It is possible that functional complementation contributes to the beneficial effects observed in this model.

Owing to the lack of neurodegeneration in *Pink1*^*−/−*^ mice, we turned to the well-established MPTP mouse model to address the following questions: first, could Drp1 inhibition prevent neurotoxicity in the nigrostriatal pathway? Second, under the scenario of pre-established loss of DA neurons as seen in PD, could Drp1 inhibition enhance DA release? As demonstrated, both AAV-mediated and small-molecule approaches conferred such beneficial effects in the MPTP model. These *in vivo* observations are also consistent with a previous *in vitro* study in which either silencing Drp1 or expressing Drp1-K38A attenuates mitochondrial dysfunction induced by MPP^+^ (ref. 17). A recent study using a complementary approach of overexpressing OPA1 also reports protection in the MPTP mouse model^49^. However, because we evaluated the protective effects of Drp1 inhibition in mice 7 days after the last MPTP injection, we cannot entirely rule out the possibility that such treatment delayed rather than prevented neurotoxicity. Future studies are needed to assess whether blocking mitochondrial fission or promoting fusion also confers neuroprotection in a longer time point post MPTP injection.

Drp1 plays an important role in the induction of cellular apoptosis pathways by regulating cytochrome *c* release. Indeed, Drp1 and the pro-apoptotic protein Bax co-localize at cleavage sites during fission^50^. Inhibition of Drp1 via pharmacological (mdivi-1)^27^ or genetic^51^ strategies exerts anti-apoptotic effects. Relevant to our data, MPTP has been demonstrated to induce cell death *in vivo* partly through Bax-dependent cytochrome *c* release^49^. Hence, because mitochondrial fission, Drp1 activity, and apoptosis are often not mutually exclusive events, it is possible that the neuroprotective effects of blocking Drp1 function in our MPTP studies are due to blocking cytochrome *c* release. Furthermore, because damage or mutations in mtDNA in nigral DA neurons have been detected after MPTP treatment^52^,^53^, blocking Drp1 may also confer protective effects through functional complementation in our studies.

Drastic alterations in either fission or fusion in a ‘normal’ neuron is not conducive to neuronal function and viability in animal models. For example, Drp1 knockout causes embryonic lethality and degeneration of Purkinje neurons in mice^54^,^55^,^56^. Similarly, deletion of the mitochondrial fusion gene *OPA1* is also embryonically lethal^57^. Although heterozygous *OPA1* mutant mice are viable, they exhibit synaptic loss^58^, a neuropathology that precedes neurodegeneration. Together, these animal models indicate that germline deletion of mitochondrial fission/fusion genes is detrimental. In contrast to the knockout mouse models above, we did not completely delete Drp1 in our study. With our Drp1-blocking strategies, it is most likely that mitochondrial fission is not entirely blocked and thus the residual effects are sufficient for the maintenance of normal physiological functions. This is a likely scenario given that only ~3% of total Drp1 is required by mitochondria for the maintenance of normal physiological functions^59^. Furthermore, we target Drp1 under pathological setting such as MPTP toxicity, which excessively enhances Drp1 function^17^. Mice systemically injected with mdivi-1 were also viable and no abnormal phenotypes were detectable. At cellular and functional levels as shown in our study (Figs 4, 5, 7), we did not detect neurotoxicity of this molecule in the brain. The effects of this molecule in the peripheral system have also recently been assessed. Using the same regimen, Mdivi-1 did not affect blood pressure, oxygen saturation, pH and blood cell counts^29^.

Mitochondrial fission is critical to mitophagy and mitochondrial motility. Cell culture studies show that blocking fission or promoting fusion impairs these processes^6^. Although this *in vitro* study cautions the potential negative impacts of altering fission/fusion, our results indicate blocking Drp1 function using our strategies does not affect synaptic release or cell viability in adult *Pink1*^*+/+*^ and regular C57BL/6 mice. Together, these studies suggest a potential difference between cell culture and animal studies. As discussed above, it is possible that mitochondrial fission is not entirely blocked by Drp1-K38A or mdivi-1 and thus the residual effects are sufficient for the maintenance of normal physiological functions. Furthermore, other studies have shown that under stressful (nutrient deprivation) or certain pathological conditions (mutant α-synuclein and huntingtin), blocking mitochondrial fission and promoting fusion protect against mitophagy^60^,^61^,^62^ and enhance mitochondrial motility^24^. The crucial role of mitochondrial fusion in the nigrostriatal system has also been recently demonstrated in a mouse model with conditional deletion of *Mfn2* in DA neurons^63^. The loss of this mitochondrial fusion gene results in progressive retrograde neurodegeneration and L-3,4-dihydroxyphenylalanine responsive locomotor deficits. Simultaneous deletion of *Mfn2* in DA neurons in the ventral tegmental area further reveals that nigral DA neurons are most vulnerable to reduced mitochondrial fusion.

Mitophagy mediated by concerted efforts of PINK1 and parkin has also been a topic of interest in recent years. Parkin is recruited by PINK1 to mitochondria with depolarized membrane potential to initiate mitophagy^64^. Most of the studies that characterize the effects of overexpressing PINK1 and Parkin on mitophagy have been performed in immortalized cells such as HeLa, MEF and SH-SY5Y cells using strong protonophores such as CCCP to collapse membrane potential. However, to date, many questions remain to be addressed. For example, what is the physiological relevance of this pathway in the mammalian brain or in the presence of endogenous levels of PINK1/Parkin? At endogenous levels, Parkin fails to mediate mitophagy in human primary fibroblasts and induced pluripotent stem cell-derived neurons^65^. Mice with mitochondrial transcription factor A deletion in dopaminergic neurons exhibit mitochondrial fragmentation, mtDNA depletion and neurodegeneration^66^; however, mitophagy was not detectable in the brain of these animals-even when Parkin was overexpressed using AAV^66^. Furthermore, when these mutant mice were crossed with Parkin-knockout mice, they did not exhibit more accumulation of damaged mitochondria and neurodegeneration^66^. Collectively, the *in vivo* significance of mitochondrial fission and PINK1 in mitophagy remains to be established.

In summary, the present study was designed to concisely address one central question: What is the functional impact of blocking or promoting fission on the nigrostriatal pathway in animal models with mitochondrial dysfunction? Our results demonstrate that blocking Drp1 function is protective against active neurotoxicity and is capable of restoring DA release under conditions of pre-existing pathology. In the latter scenario, although blocking Drp1 does not promote regeneration of DA neurons in the MPTP model with pre-established lesions (at least not within this short period of time), the combination of preventing cell loss and functional recovery of DA release in the remaining neurons is likely to be beneficial. The current cornerstone of PD treatment still remains DA replacement therapy. Given current interest on whether or in which direction promoting fusion or fission would affect synaptic release and neurodegeneration *in vivo*, we believe our results shed light on this critical issue and encourage additional future in-depth studies in this field.

## Methods

### Plasmids

*Drp1-K38A* and *Fis1* in pcDNA3 plasmids^14^ were kindly provided by Dr Yisang Yoon (Georgia Regents University). To monitor the expression of these proteins after rAAV2 injections, we tagged *Drp1-K38A* with *eGFP* and *hFis1* with *myc*, at the C-terminus using standard molecular biology techniques. Based on similar procedures as described by the Bowers Laboratory^67^, these genes were subcloned into the pBSFBRmcs shuttle vector using the following sites: *Age*I-*Bam*HI for *Drp1-K38A-eGFP* and *Bam*HI-*Eco*RI for *Fis1-myc*. The expression cassettes of these genes containing the cytomegalovirus promoter (CMV) were excised using *Not*I and then ligated (also using *Not*I) into a modified pFBGR plasmid backbone devoid of the CMV-*eGFP* expression cassette. The pFBGR plasmid harbours a CMV-driven *eGFP* gene flanked by inverted terminal repeats. This original pFBGR plasmid was used as an empty vector control for rAAV2 transduction. All pFB-related plasmids were then transiently transfected into baby hamster kidney cells and transgene expression was confirmed using immunocytochemistry before viral packaging.

### Mice

The animal procedures described in this study were approved by the University Committee on Animal Resources under the protocol # 2006-102R. All mice were housed in the animal facilities of the University of Rochester School of Medicine. Animals had free access to water and food. Veterinary care includes a full programme for prevention of disease, daily observation and surveillance for animal health, appropriate methods of disease control, diagnosis and treatment, appropriate methods of handling, restraint, anaesthesia, analgesia and euthanasia as well as monitoring of surgical programmes and post-surgical care. The generation of *Pink1*^*−/−*^ mice with a targeted germ-line deletion of exons 4–7 and a portion of exon 8 has been described^68^ and these animals have been backcrossed to C57Bl/6 background over nine to ten generations. Heterozygous breeders (*Pink1*^*+/−*^) were kept in the pathogen-free barrier area of the Micro-Isolator Facility. All *Pink1*^*−/−*^ and their *Pink1*^*+/+*^ male littermates used in this study were approximately 11- to 13-month old.

### rAAV packaging

These procedures were performed according to the previous publications from the Bowers Laboratory^67^. Briefly, rAAV2 was produced by co-infecting cultures of SF9 cells at log phase (2 × 10^6^ cells per ml) with passage 2 baculovirus of pFBDAAV (serotype viral proteins), and pFBDLSR (Rep 52 and Rep 72) and pFB- *Drp1-K38A-eGFP,* pFB-*Fis1-myc* or pFB-*eGFP* at a multiplicity of infection (MOI)=5 each. Cultures were incubated for 72 h at 28 °C and harvested by centrifugation. Pelleted cells were resuspended in PBS with MgCl_2_, serially frozen at −70 °C and thawed at 37 °C three times. The lysates were centrifuged and optical grade CsCl_2_ (Shelton Scientific) was added to supernatant; final concentration was confirmed by refractive index. rAAV particles were banded on a CsCl_2_ gradient by ultracentrifugation. Fractions with a refractive index of 1.374–1.370, corresponding to the position of viable viral particles, were collected and subsequently dialysed against PBS. AAV particles were titred, relative to rAAV-*eGFP* titres that were packaged in parallel, by transduction assay followed by flow cytometry and quantitative real-time PCR.

### Stereotactic injections of rAAV2

C57Bl/6 male mice (~10-week old) or *Pink1*^*−*/*−*^ mice and *Pink1*^*+/+*^ littermates (approximately 1-year old) received either bilateral or unilateral supranigral stereotactic injections of rAAV2 capsids. Under Avertin anaesthesia (300 mg kg^−1^), mice were positioned in a stereotactic apparatus and an incision was made to expose bregma on the skull. Burr holes were drilled over the injection coordinates (relative to bregma: −3.1 mm caudal, ±1.3 mm lateral, −4.2 mm ventral). The injection setup consisted of a frame-mounted micromanipulator, holding an UltraMicro pump (WPI Instruments) with a Hamilton syringe and a 33-GA needle (Hamilton). The needle was lowered into the parenchyma at a rate of 0.8 mm min^−1^, and then held in place for 2 min before injection. rAAV2 vectors (5 × 10^9^ transducing units) were delivered either unilateral or bilateral to the substantia nigra in a 5-μl volume using convection enhanced delivery (a method enhancing tissue volume receiving virus) by using increasing step-wise injection rates of 100 nl min^−1^ for 6 min, 200 nl min^−1^ for 10 min and 400 nl min^−1^ for 6 min. After injection, the needle was allowed to rest in place for 2 min, then withdrawn at a rate of 0.4 mm min^−1^. Incisions were sutured with 4–0 Vicryl (Ethicon, Inc.), triple antibiotic and lidocaine topical ointments were applied, and mice placed in a recovery chamber at 37 °C overnight.

### MPTP and rAAV treatments

For all studies, approximately 10- to 12-week-old male C57Bl/6 mice were randomly assigned to receive i.p. injections of MPTP (20 mg kg^−1^, Sigma) or saline once daily for 5 days. For neuroprotection studies, MPTP injections began 8 weeks after rAAV2 delivery and mice were killed 7 days after last MPTP dose. For neuro-rescue studies, rAAV2 was delivered 7 d after the last MPTP injection and mice were killed 8 weeks later. Mice were decapitated and freshly removed brains were bisected coronally ~1–2 mm caudal to optic chiasm. The caudal portion (midbrain) was immediately placed in 4% paraformaldehyde (PFA) for 24 h. The rostral portion (striata) was divided mid-sagittally. Randomly, one half was placed in 4% PFA for 24 h, whereas the other was processed for HPLC analysis. After 24 h in 4% PFA, tissue was cryoprotected in 15%, then 30% sucrose phosphate buffer, then frozen at −80 °C for immunohistochemical studies.

### Mdivi-1 preparation

Mdivi-1 (3-(2,4-dichloro-5-methoxyphenyl)-2-sulfanyl-4(3H)-quinazolinone) was purchased from Enzo Life Sciences International, Inc. and dissolved in dimethylsulphoxide (DMSO; 100 mg ml^−1^) as a stock solution. For injections, mdivi-1 was diluted in sterile saline (1% DMSO). Owing to the poor aqueous solubility of mdivi-1, each dose was gently sonicated (Model S3000 Sonicator with tapered microtip; Misonix, Inc.) at a power level 0.5–1 for 30 s producing a homogenous suspension and injected i.p. immediately. For cell culture experiments, mdivi-1 stock solution was diluted in culture medium to varying working concentrations.

### Serum and brain levels of mdivi-1

C57Bl/6 mice were injected i.p. with mdivi-1 (20 mg kg^−1^ in sterile saline containing 1% DMSO) and killed 0.5, 1, 3, 6, 12 and 24 h later. Mice were decapitated and blood was collected in heparinized tubes and centrifuged at 2,000 *g* for 10 min at 4 °C. Collected serum (100 μl) was gently vortex with acetonitrile (200 μl) for ~10 s and centrifuged at 14,000 *g* for 15 min. Supernatant was collected for HPLC analysis. Fresh brain samples were homogenized in 5 volumes (wt/vol) saline via sonication for 5–10 s. For every 100 μl homogenate, 200 μl acetonitrile was added and sonicated again to extract mdivi-1. After centrifugation at 14,000*g* for 15 min at 4 °C, supernatant was collected for HPLC analysis as described below. Half-life of mdivi-1 was calculated using the following equations: (*K*_elim_=[ln(*C*_peak_)—ln(*C*_trough_)/*T*_interval_ and *T*_1/2_=0.693/*k*_elim_).

### MPTP and mdivi-1 treatments

For all studies, 10- to 12-week-old male C57BL/6 mice were randomly assigned to receive i.p. injections of either MPTP (20 mg kg^−1^, Sigma) or saline once daily for 5 days. For the neuroprotection studies, mice received twice daily i.p. injections (20 mg kg^−1^) with mdivi-1 beginning on the day of the first MPTP injection and continued until mice were killed 7 days after last MPTP injection. For the neurorestorative studies, mice received twice daily i.p. injections (20 mg kg^−1^) with mdivi-1 beginning 7 days after the last MPTP injection and continued for a total of 3 days. To maximize the data yielded from each animal, mice were killed by decapitation and the freshly removed brains were divided into three pieces for separate measures of nigrostriatal damage. Upon removal, brains were first divided into rostral and caudal sections via a coronal cut approximately 1–2 mm caudal to the optic chiasm. The caudal portion containing the midbrain was immediately placed in 4% PFA for 24 h. The rostral portion containing the striatum was then divided mid-sagittally into right and left halves. Randomly, one half was placed in 4% PFA for 24 h, whereas the other was processed for HPLC analysis of total striatal DA. After 24 h in 4% PFA, tissue was cryoprotected in successive 15 and 30% sucrose phosphate buffer for 2 days then frozen at −80 °C for immunohistochemical studies.

### Immunohistochemistry and co-localization

Coronal brain sections (30 μm) from mice receiving rAAV2-eGFP and Drp1-K38A-eGFP were incubated in M.O.M mouse IgG blocking reagent (Vector Laboratories) overnight before incubation with polyclonal anti-GFP (1:500, Invitrogen) and monoclonal anti-TH (1:500; Sigma). For Fis1-myc, monoclonal anti-myc (1:2,000, 9B11 clone, Cell Signaling) and polyclonal anti-TH (1:500, Calbiochem) were used. Corresponding secondary antibodies Alexa Fluor 488 and 594 (Invitrogen) were used. Nuclei were visualized with 4′,6-diamidino-2-phenylindole dihydrochloride (1:1,000, Invitrogen). Images were scanned at 0.5 μm intervals throughout the whole section and analysed using confocal microscopy (FV1000; Olympus).

### Quantification of DA nigral neurons and striatal terminals

These analyses were performed as described^23^. Briefly, animals were anaesthetized with pentobarbital and intracardially perfused with cold 4% (w/v) PFA in 0.1 M PBS (pH 7.4). Their brains were removed, postfixed in the same fixative overnight at 4 °C, cryoprotected in successive 15 and 30% sucrose phosphate buffer for 2 days at 4 °C. The brains were subsequently frozen in dry ice-chilled isopentane (approximately −50 °C). Serial coronal sections (30 μm) spanning the entire midbrain and striatum were collected free-floating in PBS. Every fourth sections (for mibrain) and every eighth sections (for striatum) spanning from the caudal to rostral boundaries of these brain regions were incubated with a polyclonal anti-TH (Calbiochem) for 48 h at 4 °C. Biotinylated secondary antibodies followed by avidin-biotin complex were used. Immunoreactivity was visualized by incubation in 3,3′-diaminobenzidine/glucose/glucose oxidase. Total numbers of TH-positive neurons in SNc were counted stereologically using the optical fractionator method. Striatal optical density of TH-positive fibres was quantified using the Scion Image programme.

### Measurements of striatal MPP^+^ levels

To assess whether mdivi-1 treatment interferes with the conversion of MPTP into MPP^+^, 10- to 12-week-old male C57BL/6 mice received a single i.p. injection of mdivi-1 (20 mg kg^−1^) or vehicle followed immediately by a single i.p. injection of MPTP (25 mg kg^−1^). All mice were killed 90 min after the injections. Striatal tissue levels of MPP^+^ were measured using HPLC (CoulArray 5600A, 12-channel, ESA Inc.). Samples (20 μl) were injected manually into a Rheodyne sample injector (with a fixed loop of 20 μl) and eluted on a narrowbore column (ID: 2.1 mm, Altima HP C18 (Alltech Associates, Inc.) using mobile phases consisting of 80.5% 50 mM KH_2_PO_4_ and 9.5% acetonitrile, pH 3.2. The flow rate was set at 0.2 ml min^−1^ for all measurements.

### *In vivo* microdialysis

Stereotactic implantation of guide cannula was performed as described^69^ using the following striatal coordinates, relative to bregma: anterior–posterior +0.5 mm, lateral −2.0 mm, dorsal–ventral −1.5 mm (from surface of brain). The following day, a microdialysis probe (2-mm membrane, Bioanalytical Systems, Inc.) was inserted into the guide cannula and connected to a low torque-dual channel swivel (Instech Laboratories, Inc.), which was connected to a syringe pump perfusing with artificial cerebrospinal fluid (aCSF) at 2 μl min^−1^. After a 2-h equilibration period, dialysates were collected every 15 min for all DA release studies. Two baseline fractions were collected, after which the perfusate was switched to aCSF containing 100 mM KCl (with equimolar reduction in NaCl to maintain osmolality) for 15 min to deliver a total 240 nmol KCl, followed by a return to normal aCSF for 45 min. Afterwards, proper probe placement was verified in each animal. Serotonin and DA were measured in each sample simultaneously. Quantification of these neurotransmitters and amount of KCl delivered to striatum were calculated as described^69^. Briefly, taking into considerations of probe efficiency (~8%), flow rate (2 μl min^−1^) and duration of sample collection (15 min), values were extrapolated from the standard curves of individual compounds.

### HPLC measurements of brain DA and mdivi-1

A 12-channel CoulArray (ESA Inc.) equipped with a highly sensitive amperometric microbore cell (model 5041, ESA Inc.) was used to analyse the content of DA and its metabolites with the cell potential set at +220 mV as described^70^. For mdivi-1 pharmacokinetic studies, 20 μl samples were injected manually and separated on a narrowbore column (ID: 2.1 mm, Altima HP C18, Alltech Associates, Inc.) using a mobile phase consisting of 35 mM KH_2_PO_4_ and 45% acetonitrile, pH 3.5, and detected at 298 nm using a UV detector (model no. 526, ESA Inc.). The flow rate was set at 0.2 ml min^−1^ for catecholamines and 0.4 ml min^−1^ for mdivi-1 by using a solvent delivery pump (Model 585, ESA Inc.). Data were collected and processed using the CoulArray data analysis programme.

### Transport studies

EM4 cells, human embryonic kidney (HEK 293) cells stably transfected with macrophage scavenger to increase their adherence to tissue culture plastic^70^, overexpressing mouse^70^ DAT or empty vector control were grown in 24-well plates. These cells were washed twice and then preincubated for 20 min at 37 °C in Krebs Ringer Hepes buffer (125 mM NaCl, 25 mM HEPES, 5.6 mM glucose, 4.8 mM KCl, 1.2 mM KH_2_PO_4_, 1.2 mM CaCl_2_, 1.2 mM MgSO_4_, pH 7.4) (ref. 70), in the absence or presence of mdivi-1 (1 or 10 μM) or GBR12909 (1 μM). This buffer was then replaced with Krebs Ringer Hepes plus or minus MPP^+^ (200 μM), in the absence or presence of mdivi-1 (1 or 10 μM) or GBR12909 (1 μM, a DAT inhibitor) for 30 min. To stop the reaction, cells were rinsed with ice-cold buffer and then immediately removed in 5% trichloroacetic acid, sonicated and centrifuged at 15,000 *g* at 4 °C for a minute. Supernatant was collected for MPP^+^ quantification using HPLC as described above. Cell pellet was measured for protein concentration using the BCA assay.

### Immuno-electron microscopy

Mice were transcardially perfused with 1% glutaraldehyde/4% PFA in 0.1 M sodium cacodylate buffer, pH 7.4. Perfused brains were blocked in the coronal plane and 3 mm slices of striatum (approximately +0.7 to 0.4 mm Bregma) were removed, postfixed and then cryoprotected gradually up to 30% sucrose. Tissue was then cut into 50-μm thick coronal sections using a cryostat. Cryostat sections were treated with 1% sodium borohydride in 0.1 M Tris-buffered saline (TBS) for 30 min, washed thoroughly, then blocked in 5% Normal goat serum, 1% BSA, 0.1% cold water fish gelatin, 1% glycine and 1% lysine in 0.1 M tris-buffered saline for 1 h at room temperature. Tissue was then incubated with polyclonal anti-TH (1:100, Calbiochem) for three nights at 4 °C, followed by biotinylated goat anti-rabbit (1:200, Vector Labs) for two nights. Sections were then incubated in ExtrAvidin (1:150, Sigma) for one night at 4 °C before being reacted with 3,3′*-*diaminobenzidine, silver enhanced, gold-toned and osmicated (1% OsO_4_). Dehydrated sections were embedded in EPON/Araldite epoxy overnight, sectioned (80 nm), stained with uranyl acetate and lead citrate and examined using a Hitachi 7650 electron microscope with an attached Gatan 11 megapixel digital camera system. A blinded experimenter obtained images at 20,000 × of TH-positive terminals, of which a second blinded experimenter quantified the morphology of 50 mitochondria per mouse using ImageJ v1.42 (NIH).

### Statistics

All values are expressed as mean±s.e.m. Differences between means were analysed using a two-tailed *t*-test, one-way or two-way analysis of variance followed by Newman–Keuls *post-hoc* testing for pairwise comparison using SigmaStat v3.5. For *in vivo* microdialysis data, areas under the curve were generated using GraphPad Prism v5.01 as previously described^69^,^70^. The null hypothesis was rejected when *P*-value was <0.05.

## Additional information

**How to cite this article:** Rappold, P. M. *et al.* Drp1 inhibition attenuates neurotoxicity and dopamine release deficits *in vivo*. *Nat. Commun.* 5:5244 doi: 10.1038/ncomms6244 (2014).

## Supplementary Material

Supplementary Figures Supplementary Figure 1

## Figures and Tables

**Table 1:** Dopamine levels (pmol) in striatal dialysates collected every 15 min in rAAV2-transduced *Pink1* ^*−/−*^ and *Pink1* ^*+/+*^ mice.

**Genotype**	**rAAV2**	***n***	**0 min**	**15 min**	**30 min**	**45 min**	**60 min**	**75 min**
* Pink1 * ^*+/+*^	GFP	5	0.26±0.06	0.26±0.05	41.26±4.43	2.98±0.38	0.13±0.01	0.19±0.04
* Pink1 * ^*−/−*^	GFP	3	0.19±0.03	0.22±0.02	17.55±3.07	2.06±0.18	0.19±0.06	0.11±0.01
* Pink1 * ^*+/+*^	K38A	4	0.22±0.04	0.25±0.05	42.21±3.89	3.53±0.88	0.18±0.03	0.13±0.05
* Pink1 * ^*−/−*^	K38A	4	0.33±0.07	0.28±0.07	42.83±1.87	5.65±0.78	0.19±0.06	0.13±0.02
* Pink1 * ^*+/+*^	Fis1	4	0.22±0.03	0.24±0.02	29.45±2.40	2.62±0.33	0.19±0.03	0.15±0.03
* Pink1 * ^*−/−*^	Fis1	3	0.19±0.04	0.14±0.04	19.46±1.33	1.65±0.14	0.18±0.05	0.13±0.03

GFP, green fluorescent protein; rAAV, recombinant adeno-associated virus.

**Table 2:** Dopamine levels (pmol) in striatal dialysates collected every 15 min in mdivi-1-treated-*Pink1* ^*−/−*^ and *Pink1* ^*+/+*^ mice.

**Genotype**	**Treatment**	***n***	**0 min**	**15 min**	**30 min**	**45 min**	**60 min**	**75 min**
* Pink1 * ^*+/+*^	Vehicle	5	0.47±0.07	0.40±0.09	36.11±3.68	5.58±1.52	0.87±0.19	0.49±0.09
* Pink1 * ^*−/−*^	Vehicle	4	0.44±0.08	0.53±0.14	15.24±3.44	1.39±0.31	0.36±0.06	0.30±0.05
* Pink1 * ^*−/−*^	Mdivi-1	4	0.53±0.10	0.47±0.07	38.97±6.53	4.99±0.80	1.19±0.21	0.62±0.10

**Table 3:** Dopamine levels (pmol) in striatal dialysates collected every 15 min in MPTP pre-lesioned mice with Drp1 inhibition.

**Treatment**	**Drp1 inhibition**	***n***	**0 min**	**15 min**	**30 min**	**45 min**	**60 min**	**75 min**
*a*								
Saline	GFP	5	1.27±0.22	1.17±0.39	95.35±5.67	5.62±2.07	1.08±0.39	1.04±0.37
MPTP	GFP	4	2.39±0.06	2.24±0.19	36.04±4.83	4.32±0.55	2.08±0.06	1.98±0.02
Saline	K38A	5	2.01±0.07	2.01±0.01	98.59±8.96	6.38±2.65	2.11±0.06	1.92±0.02
MPTP	K38A	4	2.08±0.06	2.12±0.07	58.17±5.83	4.51±0.71	2.05±0.04	2.04±0.04
								
*b*								
Saline	Vehicle	5	2.71±0.23	2.98±0.17	66.25±3.15	13.28±2.28	3.36±0.36	2.59±0.10
Saline	Mdivi-1	4	2.80±0.21	2.68±0.07	60.01±13.6	8.28±2.21	2.76±0.15	2.48±0.07
MPTP	Vehicle	5	2.88±0.17	2.78±0.16	20.10±3.46	6.01±0.99	2.79±0.19	2.41±0.06
MPTP	Mdivi-1	5	3.73±0.36	3.40±0.55	39.45±7.11	10.95±1.74	2.88±0.08	2.81±0.14

GFP, green fluorescent protein.
