# Supplementary material for: Drp1 inhibition attenuates neurotoxicity and dopamine release deficits in vivo
Source: Nat Commun. 2014 Nov 5;5:5244. doi: 10.1038/ncomms6244 (PMC4223875; doi:10.1038/ncomms6244)
Supplement: Supplementary Figures — Supplementary Figure 1 [file ncomms6244-s1.pdf]

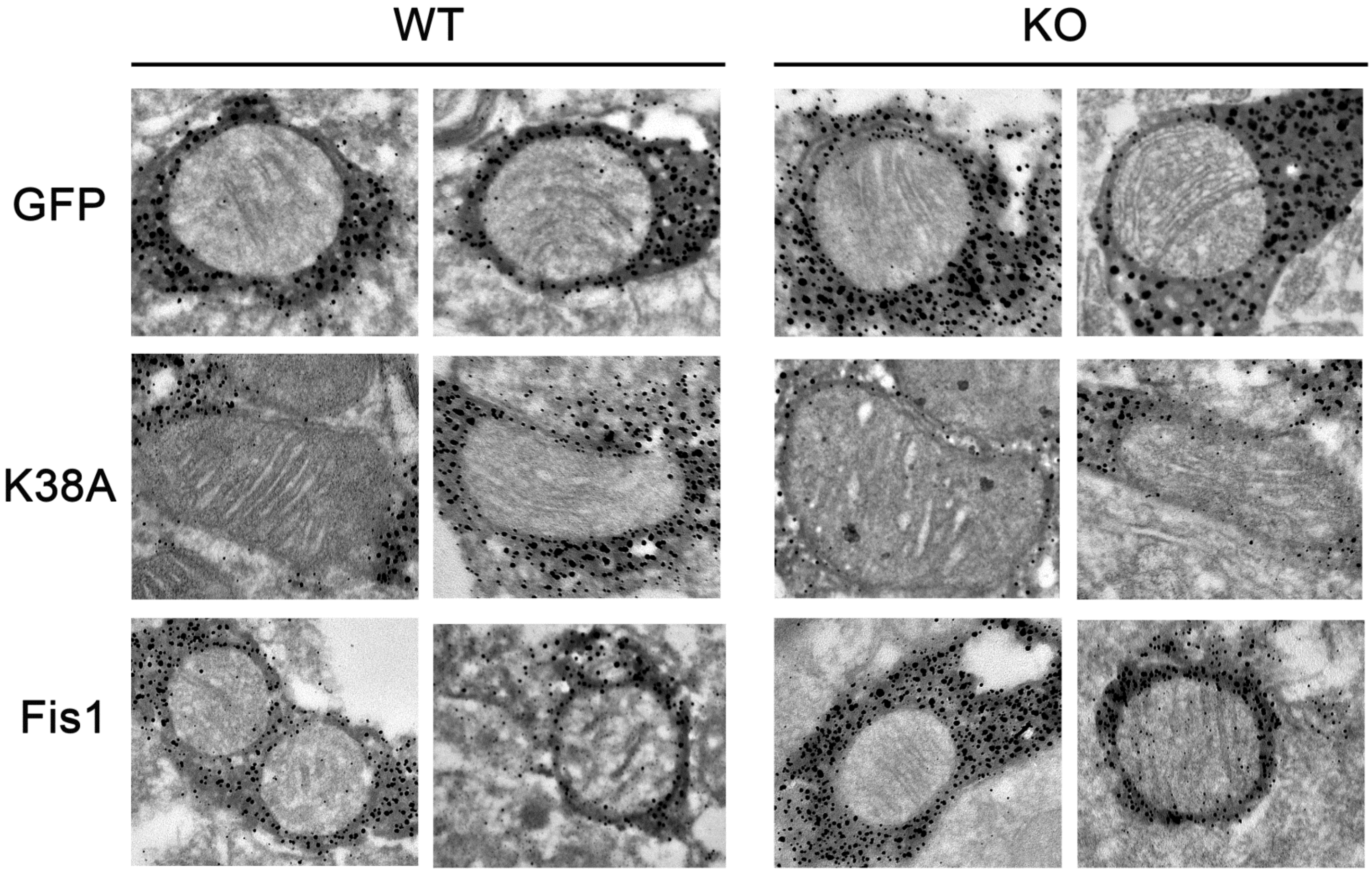

Supplementary Figure 1. Ultrastructures of mitochondria in striatal dopaminergic axonal terminals. Immuno-electron microscopy using tyrosine hydroxylase as a phenotypic marker for dopaminergic terminals was performed in coronal striatal sections from ~ 1-year old *Pink1*$^{+/+}$ (WT) and *Pink1*$^{-/-}$ (KO) littermates transduced with rAAV2-eGFP control, rAAV2-Drp1-K38A, or rAAV2-Fis1.
